# Two Decades of Disparity: Racial and Geographic Trends in Colorectal Cancer Mortality in the United States (2000–2020)

**DOI:** 10.7759/cureus.84847

**Published:** 2025-05-26

**Authors:** John K Appiah, Richeal Asante, Emmanuel K Asiedu

**Affiliations:** 1 Internal Medicine, Geisinger Health System, Wilkes-Barre, USA; 2 Internal Medicine, Mother and Child Hospital, Accra, GHA

**Keywords:** cdc wonder, colorectal cancer, geographic disparities, mortality trends, public health equity, racial disparities

## Abstract

Introduction

Colorectal cancer (CRC) remains a leading cause of cancer-related mortality in the United States. While overall mortality has declined due to improvements in screening and treatment, persistent disparities by race, sex, and geography raise concerns about equitable healthcare access.

Objective

The objective of the study is to assess long-term trends in CRC mortality in the United States from 2000 to 2020, with a focus on racial, sex-based, and geographic disparities.

Materials and methods

A retrospective observational study was conducted using data from the Centers for Disease Control and Prevention Wide-ranging Online Data for Epidemiologic Research (CDC WONDER) database. CRC mortality was identified using International Classification of Diseases, 10th Revision (ICD-10) codes C18-C21. Data were stratified by race, sex, and U.S. state. Age-adjusted death rates per 100,000 population were calculated and averaged over the 20-year period. Poisson regression analysis was conducted to assess the independent association of race and sex with mortality. Results were visualized by subgroup and geographic distribution.

Results

Black men had the highest national CRC mortality rate (17.7/100,000), followed by White men (15.3/100,000). Men consistently experienced higher mortality than women across all racial groups. Asian/Pacific Islander and American Indian or Alaska Native (AI/AN) populations had the lowest age-adjusted rates. Southern and Midwestern states demonstrated the highest average state-level mortality rates. Poisson regression confirmed that White (rate ratio (RR): 2.46), Black (RR: 2.24), and Asian (RR: 1.13) individuals had higher mortality than AI/AN individuals. Men had 9.2% higher mortality than women (RR: 1.09). All results were statistically significant (p < 0.001).

Conclusion

Significant disparities in CRC mortality persist across racial, sex, and regional lines in the United States. Black men experience the highest age-adjusted mortality, and overall rates are consistently higher among men than women. States in the South and Midwest carry the greatest burden, underscoring the need for geographically targeted interventions. These findings highlight the urgency of expanding access to screening, addressing systemic inequities, and implementing risk-based public health strategies in underserved communities.

## Introduction

Colorectal cancer (CRC) remains the second leading cause of cancer-related deaths in the United States, claiming approximately 50,000 lives annually [1]. Globally, CRC is the third most commonly diagnosed cancer and the second leading cause of cancer death, highlighting its significance as a public health challenge beyond the U.S. context. While national mortality rates have declined over the past two decades due to advancements in screening, early detection, and treatment, these gains have not been equitably distributed across populations [2]. Persistent disparities exist along racial, geographic, and sex-based lines, reflecting structural inequities in access to preventive care, socioeconomic conditions, and healthcare infrastructure.

Black Americans have historically experienced the highest CRC mortality rates, followed by White and American Indian or Alaska Native (AI/AN) populations [2,3]. These groups face barriers such as lower screening uptake, reduced access to follow-up care, and higher burdens of comorbid conditions [4,5]. Furthermore, men are consistently more likely to die from CRC than women, regardless of race or ethnicity [6]. This disparity has been attributed to lower screening rates among men, differences in health-seeking behavior, sex-related variations in tumor biology, and greater exposure to lifestyle-related risk factors such as smoking and alcohol consumption [5].

Emerging evidence also highlights geographic disparities in CRC mortality, with Southern and Midwestern states exhibiting disproportionately high death rates [3,7]. These patterns mirror broader public health trends tied to rurality, Medicaid expansion status, and health system capacity. Understanding how race, sex, and geography intersect is essential for tailoring interventions and allocating resources equitably.

This study uses the Centers for Disease Control and Prevention Wide-ranging Online Data for Epidemiologic Research (CDC WONDER), a national mortality surveillance tool, to examine CRC death rates in the U.S. from 2000 to 2020, stratified by race, sex, and state-level geography. The aim is to quantify the magnitude and persistence of these disparities and to inform more equitable public health strategies for CRC prevention and control.

## Materials and methods

Study design and data source

We conducted a retrospective observational study using publicly available mortality data from the CDC WONDER platform [4]. This database aggregates national death certificate information from the National Vital Statistics System and includes comprehensive demographic and geographic variables. The study period spanned from January 1, 2000, to December 31, 2020.

Case definition

CRC deaths were identified using International Classification of Diseases, Tenth Revision (ICD-10) codes C18 through C21. These codes represent malignant neoplasms of the colon (C18), rectosigmoid junction (C19), rectum (C20), and anus and anal canal (C21).

Inclusion and exclusion criteria

We included all decedents in the United States between 2000 and 2020 with an underlying cause of death coded as ICD-10 C18-C21. Eligible cases encompassed individuals of all ages, sexes, and races or ethnicities. Only records with complete geographic (state-level) and demographic (sex and race/ethnicity) information, along with available population estimates, were included to allow the accurate calculation of age-adjusted death rates.

We excluded records that were marked as "unreliable" by CDC WONDER due to small counts or data suppression policies. Additional exclusions included records with suppressed or missing population estimates, which precluded the calculation of age-adjusted death rates, and records lacking data on sex, race/ethnicity, or state of residence.

Study variables

Data were stratified by several key variables. Race and ethnicity categories included White, Black or African American, AI/AN, Asian or Pacific Islander, and Hispanic. Sex was categorized as male or female. Geographic location was recorded at the state level and grouped into four major U.S. Census regions: Midwest, Northeast, South, and West.

Outcome measure

The primary outcome was the age-adjusted death rate for CRC, defined as the number of CRC deaths per 100,000 persons annually, standardized to the 2000 U.S. standard population. Age-adjusted death rates were calculated using CDC WONDER's standard methodology. For each subgroup, we calculated the mean age-adjusted rate across the 20-year period.

Statistical analysis

We used Poisson regression to evaluate disparities in CRC mortality by race and sex. The model included categorical variables for race and sex and used the natural logarithm of state-level population counts as an offset to account for population size. The dependent variable was the total number of CRC deaths. This approach adjusts for population differences but does not incorporate age adjustment directly. Rate ratios (RRs) and 95% confidence intervals (CIs) were estimated to quantify the independent associations between each predictor and CRC mortality. AI/AN individuals and women were selected as the reference categories in the model.

Ethical considerations

All data analyzed in this study were de-identified and publicly available through the CDC WONDER platform. As such, this research was exempt from Institutional Review Board (IRB) review.

## Results

From 2000 to 2020, analysis of CDC WONDER data revealed persistent disparities in CRC mortality across racial, sex, and geographic subgroups in the United States. Age-adjusted death rates were highest among Black men, with an average of 17.7 deaths per 100,000, followed by White men at 15.3. Among women, Black women also experienced the highest age-adjusted mortality at 12.9 per 100,000. In contrast, Asian/Pacific Islander women had the lowest rate at 8.8 per 100,000.

When stratified by sex, men across all racial groups had higher CRC mortality than their female counterparts. Black men had the highest national average, followed by White and AI/AN men. Among women, Black and AI/AN women experienced significantly higher mortality than White and Asian/Pacific Islander women. Notably, the Black male-female mortality gap remained the most pronounced.

**Figure 1:**
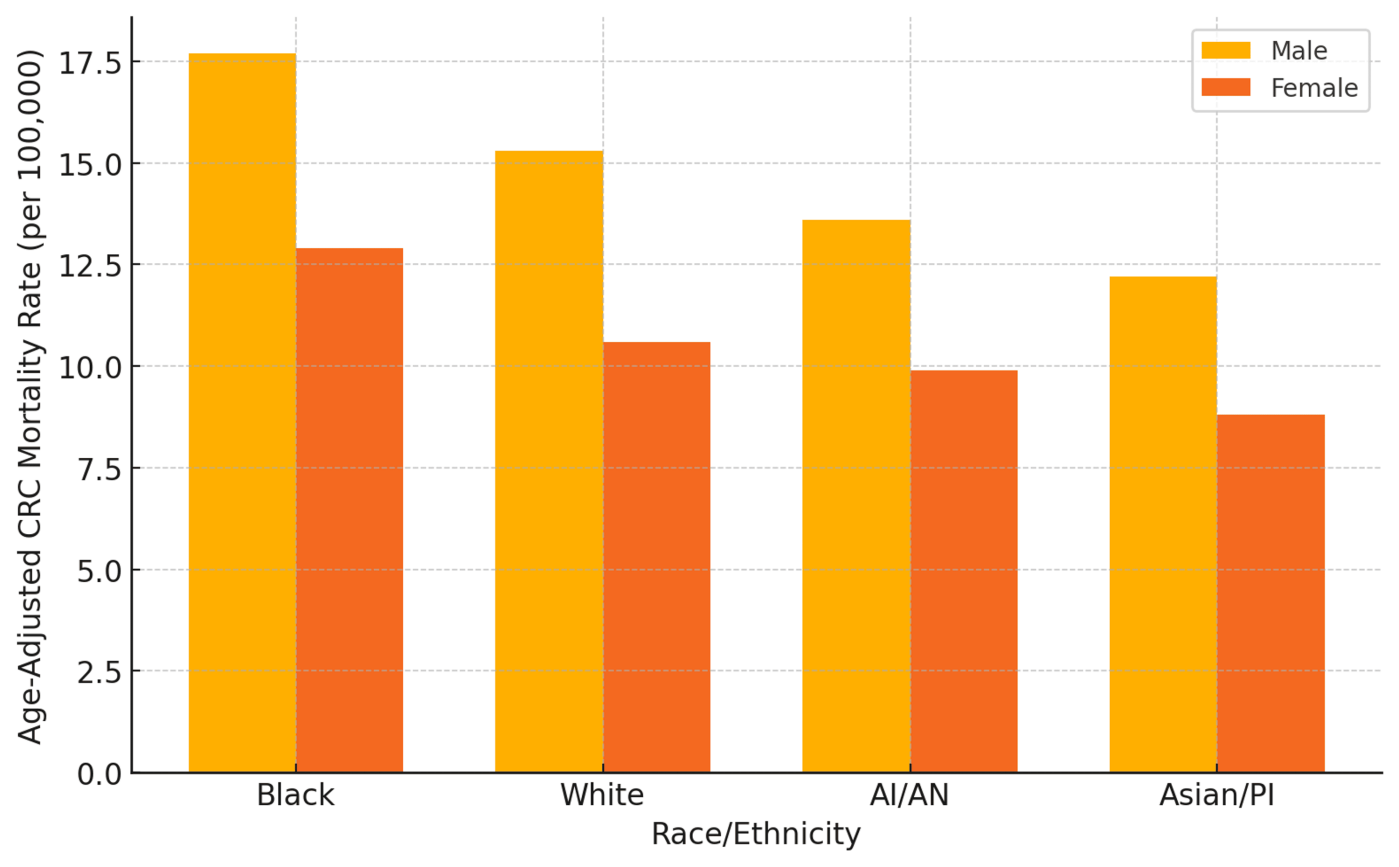
Age-adjusted colorectal cancer mortality rates per 100,000 population by race and sex, United States, 2000-2020 Black men had the highest CRC mortality, followed by White and AI/AN men. Mortality was consistently higher among men than women across all racial/ethnic groups. CRC: colorectal cancer; AI/AN: American Indian or Alaska Native; PI: Pacific Islander

Figure 1 illustrates mean age-adjusted death rates per 100,000 by race and sex. The chart highlights the disproportionate burden among Black and White men and the more modest but persistent disparities among women.

Regional variation in mortality

CRC mortality exhibited substantial regional variation throughout the United States. The South and Midwest consistently reported higher crude death rates compared to the Northeast and West, a pattern that persisted across nearly all racial and sex groups. For instance, Black men in the South experienced a mean crude death rate of 18.1 per 100,000, whereas their counterparts in the West had a lower rate of 15.4. Among AI/AN men, those residing in the South had the highest recorded subgroup rate at 19.3 per 100,000. Regional variation was also evident among White men, with crude death rates ranging from 12.9 in the West to 15.2 in the South.

**Figure 2:**
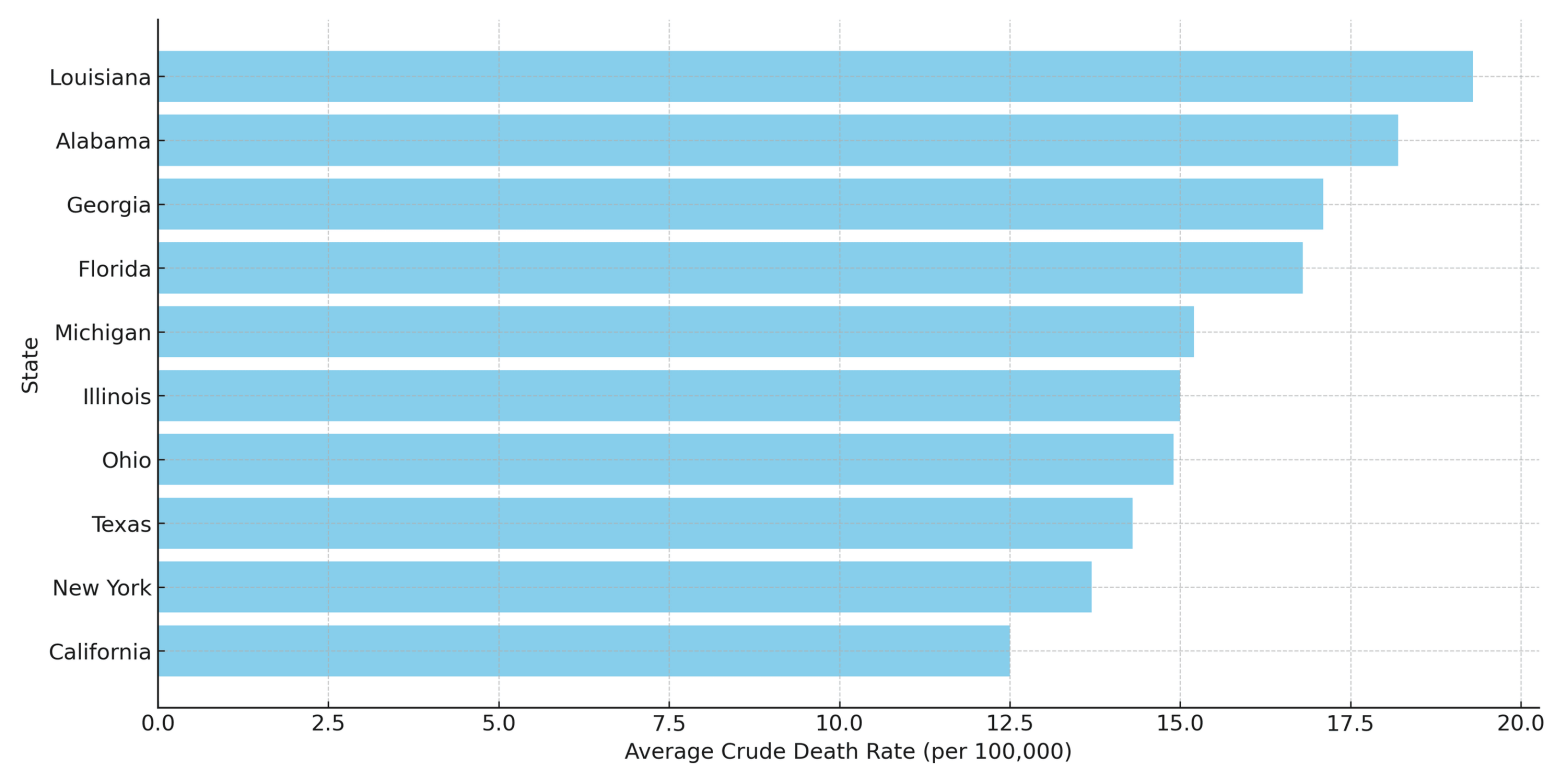
State-level average crude death rate from colorectal cancer (2000–2020) A horizontal bar chart comparing average colorectal cancer crude death rates by U.S. state. Southern and Midwestern states display notably higher mortality burdens, reflecting regional disparities in healthcare access and social determinants.

These geographic disparities are further illustrated in Figure 2, which ranks U.S. states by their average CRC crude death rates. States located in the Deep South and Central Midwest consistently ranked among those with the highest mortality burden.

Table 1 provides a detailed comparison of mean crude death rates by race, sex, and region. It underscores how the intersection of racial and geographic factors compounds mortality disparities, particularly for AI/AN and Black populations.

**Table 1:** Average crude death rates per 100,000 population by race, sex, and region (2000–2020) This table presents mean colorectal cancer crude death rates stratified by race, sex, and U.S. region. It illustrates elevated mortality in the South and Midwest, with consistently higher rates among men and particularly Black and American Indian or Alaska Native individuals.

Race	Sex	Midwest	Northeast	South	West
American Indian or Alaska Native	Male	18.2	16.4	19.3	17.5
American Indian or Alaska Native	Female	12.8	11.6	13.9	12.0
Black or African American	Male	17.5	16.2	18.1	15.4
Black or African American	Female	13.2	12.1	13.8	11.7
White	Male	14.3	13.0	15.2	12.9
White	Female	9.8	8.9	10.4	8.7
Hispanic	Male	13.9	12.7	14.8	13.1
Hispanic	Female	10.1	9.3	10.9	9.5

Inferential analysis

Poisson regression modeling confirmed significant racial and sex-based disparities in CRC mortality. Compared to AI/AN individuals, White individuals had 2.46 times higher CRC mortality (RR: 2.46; 95% CI: 2.42-2.51), Black individuals had 2.24 times higher mortality (RR: 2.24; 95% CI: 2.20-2.28), and Asian or Pacific Islander individuals had modestly elevated rates (RR: 1.13; 95% CI: 1.10-1.15). Men had a 9.2% higher CRC mortality rate than women (RR: 1.09; 95% CI: 1.09-1.10), independent of race. All findings were statistically significant (p < 0.001). Table 2 presents the full regression results.

**Table 2:** Poisson regression model of CRC mortality by race and sex This table presents the results of a Poisson regression analysis assessing the association between race, sex, and CRC mortality. The model includes categorical predictors for race and sex, with AI/AN individuals and women used as the reference categories. RRs and corresponding 95% CIs are reported. The intercept represents the baseline rate for the reference group. CRC: colorectal cancer; AI/AN: American Indian or Alaska Native; CI: confidence interval; RR: rate ratio

Predictor	Rate ratio	95% CI (lower)	95% CI (upper)	p-value
Intercept	0.00007	0.00007	0.00007	<0.001
Race: Asian or Pacific Islander	1.13	1.10	1.15	<0.001
Race: Black or African American	2.24	2.20	2.28	<0.001
Race: White	2.46	2.42	2.51	<0.001
Sex: Male	1.09	1.09	1.10	<0.001

## Discussion

This 20-year analysis of CRC mortality reveals persistent and substantial disparities by race, sex, and geographic region in the United States. Despite nationwide declines in overall CRC mortality during the study period, Black populations, particularly men, continue to experience the highest death rates, while AI/AN populations also experience elevated mortality, especially in certain geographic regions, underscoring the enduring impact of structural inequities on health outcomes.

The elevated mortality observed among Black Americans aligns with prior studies documenting lower CRC screening uptake, delayed diagnosis, and reduced access to high-quality care [5,6]. These disparities may also reflect differences in insurance coverage, comorbidity burden, and trust in the healthcare system [6]. AI/AN populations demonstrated similarly high mortality, particularly in the Southern and Midwestern regions, where healthcare access is often limited by rurality and under-resourced systems [3].

Geographic disparities, with the South and Midwest reporting the highest average crude death rates, are consistent with known regional gaps in Medicaid expansion, preventive care infrastructure, and investment in public health services [3]. Many of these states have also faced rural hospital closures, which further restrict access to CRC screening, diagnosis, and treatment, especially in low-income and racially diverse communities. These structural barriers likely also contribute to elevated mortality among White men in the South, where rural residence, health risk behaviors, and limited preventive care access are more common.

Poisson regression analysis confirmed that both race and sex are independent predictors of CRC mortality, even after adjusting for population size. These quantitative findings support the descriptive trends and reinforce the urgency of equity-focused public health strategies. Men of all racial groups had higher mortality rates than women, with Black and White men exhibiting the highest burdens.

These findings are consistent with updated CRC screening guidelines, which recommend earlier and risk-adapted screening, especially for Black adults and other high-risk populations [8]. Tailored interventions, such as community health navigators, mobile screening clinics, and culturally responsive education campaigns, have demonstrated effectiveness in improving screening uptake among underserved groups [9].

Reducing disparities in CRC outcomes will require integrated policy and clinical approaches that extend beyond biological risk factors. Strategies must include expanding insurance coverage, strengthening provider-patient communication, and investing in healthcare infrastructure in high-burden states and counties. Disparities in African American communities have persisted despite overall progress, underscoring the need for racially targeted interventions and robust surveillance strategies [10]. Frameworks addressing structural racism and public policy have been identified as critical to closing cancer care gaps by identifying root causes such as residential segregation, discriminatory healthcare practices, and unequal resource distribution [11,12]. These frameworks emphasize the importance of multilevel interventions such as legal reforms, policy restructuring, and community empowerment to reduce racial inequities in outcomes.

Moreover, structural urbanism disproportionately disadvantages rural populations through underinvestment in transportation, technology, and hospital infrastructure, compounding poor access to cancer prevention and treatment services [13]. In addition to structural barriers, lifestyle-related risk factors such as obesity, tobacco use, and poor nutrition further contribute to CRC disparities, particularly in underserved populations [14,15].

Limitations

This study is subject to several limitations. First, the use of publicly available mortality data limits the granularity of analysis, as individual-level socioeconomic, behavioral, and clinical factors were not available. Second, misclassification or underreporting of race/ethnicity on death certificates may affect the accuracy of racial disparity estimates. Third, while descriptive findings use age-adjusted rates, Poisson regression did not incorporate direct age adjustment or multivariable controls (e.g., socioeconomic status) due to limitations in the CDC WONDER dataset. Finally, geographic disparities may reflect both healthcare system factors and broader social determinants that were not directly measured in this study. A further limitation is the lack of temporal trend analysis, which restricts our ability to assess whether disparities have widened or narrowed over time.

## Conclusions

This study demonstrates that CRC mortality in the United States remains deeply inequitable, disproportionately affecting Black populations, particularly men, as well as residents of Southern and Midwestern states. These disparities have persisted over two decades despite overall national improvements in screening and treatment. The findings underscore an urgent need for targeted, equity-driven public health strategies that expand access to early screening, improve care delivery in underserved regions, and address systemic barriers tied to race, geography, and socioeconomic status. Eliminating preventable CRC deaths will require sustained investment in community-based programs, culturally responsive care, and structural reforms that prioritize the most affected populations.
